# Colloidal solution of silver nanoparticles for label-free colorimetric sensing of ammonia in aqueous solutions

**DOI:** 10.3762/bjnano.9.48

**Published:** 2018-02-09

**Authors:** Alessandro Buccolieri, Antonio Serra, Gabriele Giancane, Daniela Manno

**Affiliations:** 1 GFA-Gruppo di Fisica Applicata, Dipartimento di Matematica e Fisica “E. De Giorgi”, Università del Salento Lecce, Italy

**Keywords:** electron diffraction, electron microscopy, NH_3_ sensors, silver nanoparticles, UV–vis spectroscopy

## Abstract

Silver nanoparticles were synthesized in the presence of saccharides and ammonia (NH_3_) in the concentration range from 10^−2^ to 10^3^ ppm to develop an optical sensor for NH_3_ in aqueous solutions. Ammonia affects the features of the nanoparticles obtained in a concentration-dependent manner as determined by UV–vis absorption analysis and TEM observations. Structural and morphological analysis provides the basis for the production of a colorimetric label-free sensor for ammonia. Overall, surface plasmon resonance increases when ammonia concentration rises, although the functional trend is not the same over the entire investigated ammonia concentration range. Three different ranges have been identified: very low ammonia concentrations from 0.01 to 0.2 ppm, high ammonia concentrations from 20 to 350 ppm and, most importantly, the intermediate or physiological range of ammonia from 0.5 to 10 ppm.

## Introduction

Important sources of ammonia include synthetic fertilizers, oceans, the burning of biomass, the decomposition of plants, natural land [1] and the chemical industry [2]. Ammonia is also an organic compound normally produced by human metabolism [3] through the urea cycle [4]. Hyperammonemia (high blood levels of ammonia) is associated with kidney or liver dysfunction [5], but also with stress conditions, such as sport performances, where levels of ammonia in the blood can increase and exceed the concentration of ammonia in the air [6]. This is related to the re-metabolization of creatinine and urea into ammonia, which results in an abnormal increase of ammonia levels in the blood [7]. It is evident that early diagnosis is crucial, not only to improve prognosis, but also, to prevent the development of more serious illnesses.

The monitoring of volatile molecules in a fluid can be efficiently solved by developing methods for the detection of chemical species in the fluid itself. This approach has several advantages, including minimal fluid management and therefore greater reliability of the result.

There are many analytical methods to detect ammonia. Electrochemical analysis [8], gas chromatography [9], immunoassays [10], thin-layer chromatography [11], high-performance liquid chromatography (HPLC) [12] are the main methods used so far for a reliable determination of NH_3_ levels. However, the above-mentioned techniques have difficulties and disadvantages, such as the need for electrodes, the development of surface potentials and the volatility of ammonia. Some of the methods are relatively slow and/or require expensive equipment. Therefore, it is an urgent need to develop a rapid, accurate, simple and inexpensive method to detect NH_3_.

Metal nanoparticles, and in particular silver nanoparticles (AgNPs), are often considered for analytical application because of their peculiar optical and electrical properties [13]. The surface plasmon resonance (SPR) properties of metal nanoparticles are considered very useful for the use of colloidal solutions in the field of sensors [14]. The optical properties of gold and silver nanoparticles in the UV–vis region (200–800 nm) are well known. As an example, the peak of the optical absorption SPR is a feature of the colloidal metal nanoparticle solutions. The exact position of the absorption maximum and the shape of the peak are strongly dependent on the size, shape and interparticle distance of the nanoparticles, but also on the surrounding environment and on the number of interacting nanoparticles [15]. The optical properties of nanoparticles have allowed researchers to develop new diagnostic methods that are potentially useful for colorimetric measurements [16]. A colloidal solution of silver nanoparticles was developed for a colorimetric sensor for triethylamine [17].

So far, many authors reported the effect of ammonia on the synthesis of silver nanoparticles in colloidal solutions. In particular, it is possible to control and stabilize the synthesis of nanoparticles by adding ammonia in colloidal solutions [18]. It is also known that ammonia reacts with silver ions and gives rise to [Ag(NH_3_)_2_]^+^ [19–21], a weak oxidant able to decrease the reduction rate [22]. When very stable complexes between silver cations and ammonia anions are formed, the formation of Ag nanoparticles is inhibited. Recent results seem to disagree with previous reports about the role of ammonia and show an increase in the plasmon resonance intensity of silver nanoparticles synthesized in the presence of ammonia [23–24]. On the other hand, the presence of glucose acting as reducing agent promotes the synthesis of silver nanoparticles [25]. The addition of ammonia can change the kinetics of the synthesis of silver nanoparticles improving oxidation of hydroxy groups in glucose, and giving rise to an accelerated reduction of silver in the solution (2Ag^+^ + R_2_CH–OH + 2OH^−^ → R_2_C=O + 2H_2_O + 2Ag ), in agreement with Muench et al. [26].

Nesakumar et al. have recently synthesized silver nanoparticles (AgNPs) using Terminalia chebula extract [27] and have obtained an optical sensor to detect dissolved ammonia in water. The above mentioned work concludes with the interesting result that SPR intensity and ammonia concentration are linearly related in the investigated range from 0 (control) to 100 ppm.

In this paper we have analysed the role of ammonia in the synthesis of silver nanoparticles, a controversial topic that has not been clarified completely in the works published so far. As the results show a dependence of the plasmon resonance on the concentration of NH_3_, we propose the development of a NH_3_ “fluid” optical sensor. It is worth to stress that the analysed system, ammonia in a colloidal silver solution is very simple compared to physiological systems of ammonia in organic fluids (blood or urine). However, this study is preliminary to address the problem of interfering species, e.g., proteins, enzymes and mineral salts. We highlighted that colloidal AgNPs solutions synthesized using both glucose and sucralose as reducing and capping agents, change their colour from pale yellow to orange and exhibit increased SPR when the NH_3_ concentration increases. The accurate morphological and structural analysis of colloidal solutions obtained explains the interaction of silver particles with ammonia.

Basically this work describes a fast, reliable and low-cost strategy to detect the presence of dissolved ammonia in fluids in the wide range of concentrations from 10^−2^ to 10^3^ ppm. To better emphasize the peculiarities of different levels of ammonia, the analysed range is subdivided into three different ranges: low ammonia concentrations ranging from 0.01 to 0.2 ppm, high concentrations of ammonia ranging from 20 to 350 ppm and especially intermediate or physiological ammonia concentrations ranging from 0.5 to 10 ppm.

## Results and Discussion

**Figure 1:**
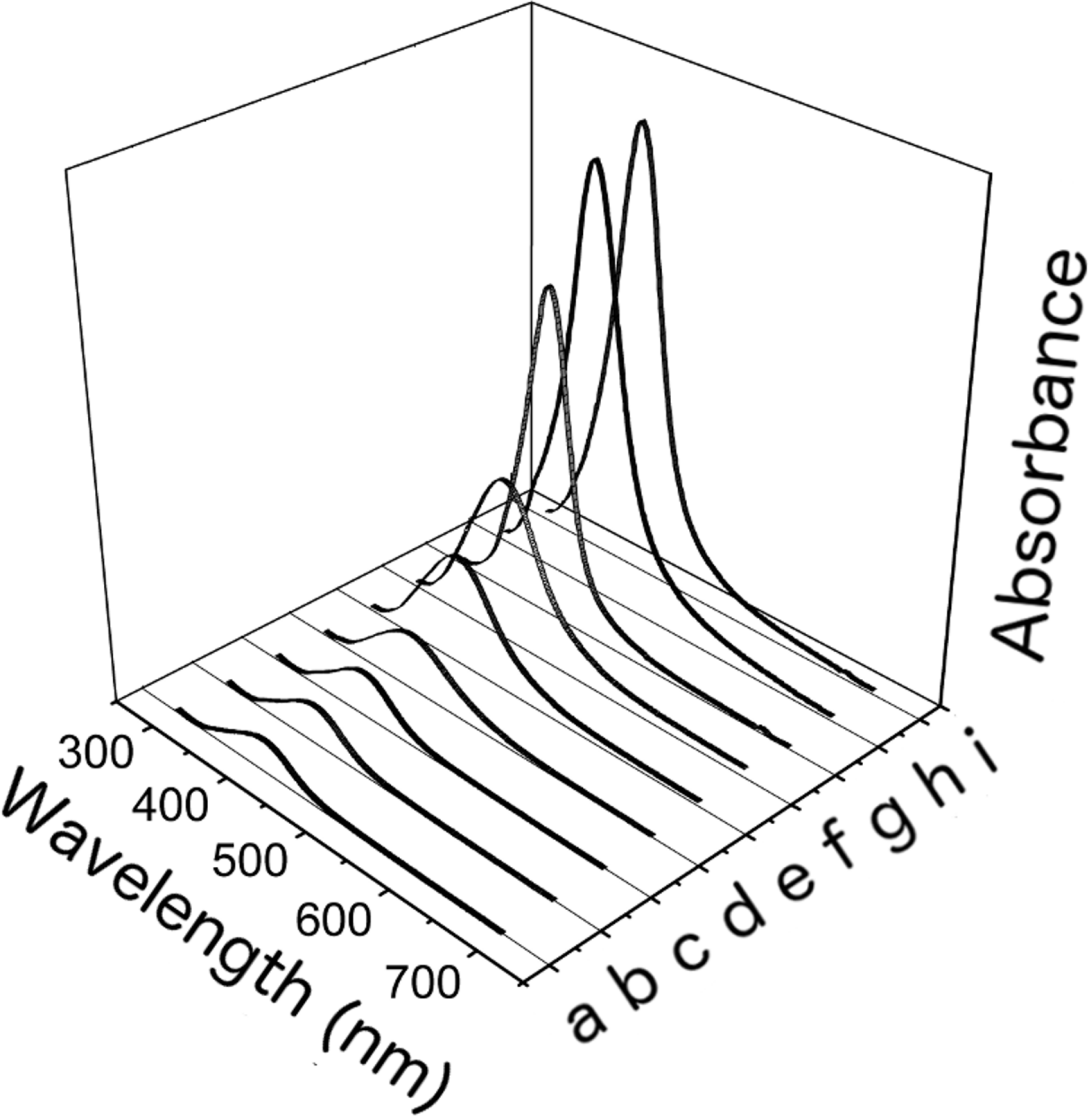
UV–vis absorption spectra obtained from (a) the colloidal solution without ammonia and (b–i) from solutions containing 0.01, 0.1, 1, 5, 10, 100, 1000 and 2000 ppm ammonia.

To analyse the role of ammonia on the optical properties of colloidal solutions of silver nanoparticles, we show in Figure 1 the UV–vis absorption spectra obtained from the most representative samples.

The peak at about 400 nm is due to SPR of the electrons in the conduction band of silver and indicates the formation of silver colloids with nanometre-sized dimensions.

Ammonia modulates the UV–vis absorption spectra in a concentration-dependent manner and catalyses the formation of silver nanoparticles. Size, shape and surface functionalization of the nanoparticles can strongly influence the spectral trend of the plasmon band of the silver nanoparticles [28–29]. As evident from Figure 1, the plasmon peak profile does not change while the ammonia concentration increases, except for very low ammonia concentrations as it will be discussed below. This observation prompted us to consider only the nanoparticle concentration as the cause of the intensity change of the plasmon band of the Ag nanoparticles. As can be seen from Figure 1, SPR increases in height, becomes wider and moves to higher wavelengths as NH_3_ concentration increases from 0 to 1 ppm. The further increase in NH_3_ concentration (from 1 to 100 ppm) causes a significant reduction of SPR width and an increase in intensity. After that a saturation process occurs as the ammonia concentration reaches 1000 ppm.

**Figure 2:**
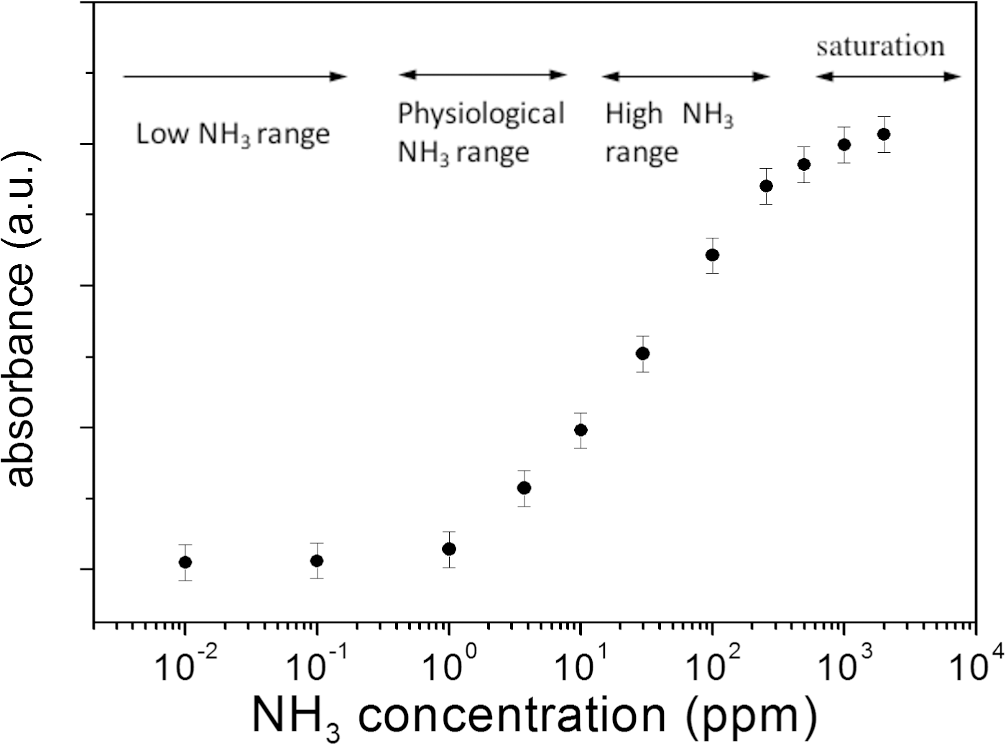
Maximum value of absorbance as a function of the NH_3_ concentration.

Ammonia influences both the number and the morphological properties of silver nanoparticles. For a better description of the optical properties of silver nanoparticles, it is useful to plot the SPR intensity as a function of the NH_3_ concentration as shown in Figure 2. The absorbance intensity increases with the NH_3_ concentration and saturates when the concentration exceeds 100 ppm.

**Figure 3:**
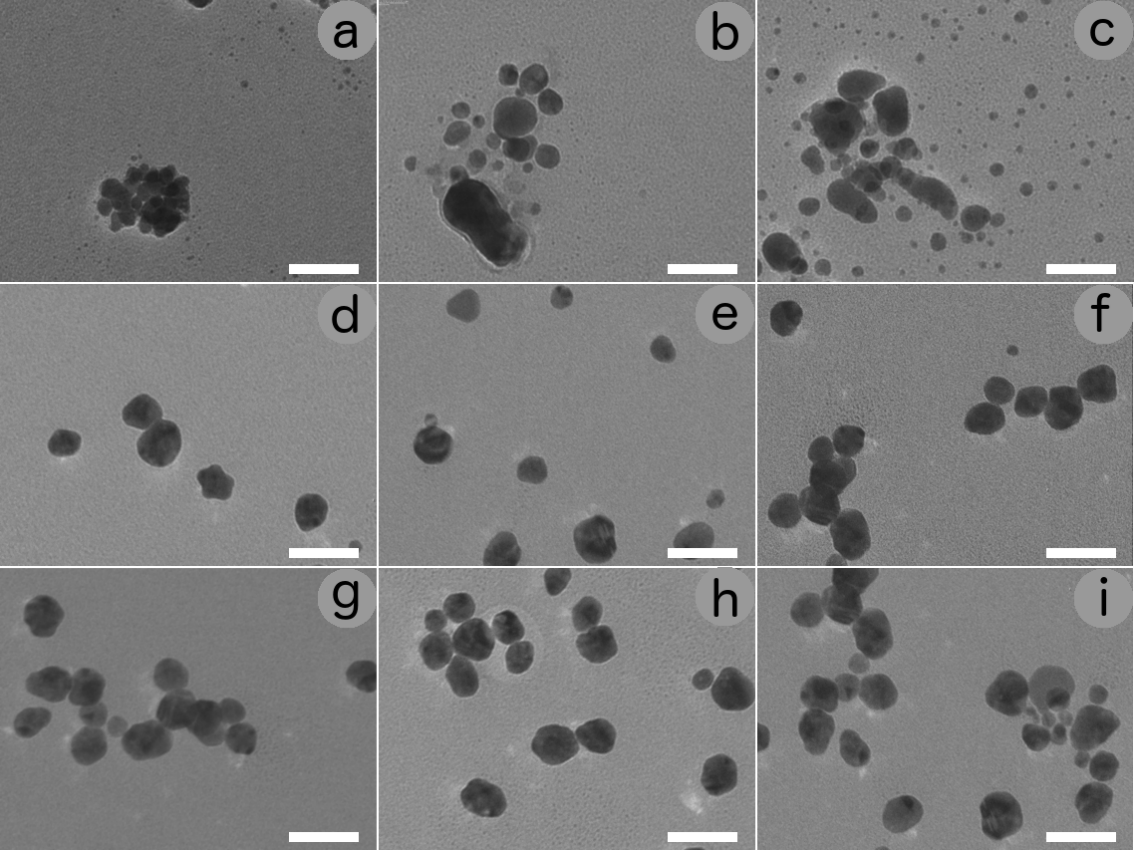
TEM images of AgNPs obtained at different NH_3_ concentrations: (a) without ammonia, and (b–i) with 0.01, 0.1, 1, 5, 10, 100, 1000 and 2000 ppm ammonia. Scale bar: 50 μm.

Figure 3 shows the TEM images recorded on different samples with ammonia concentrations ranging from 0 to 2000 ppm.

**Figure 4:**
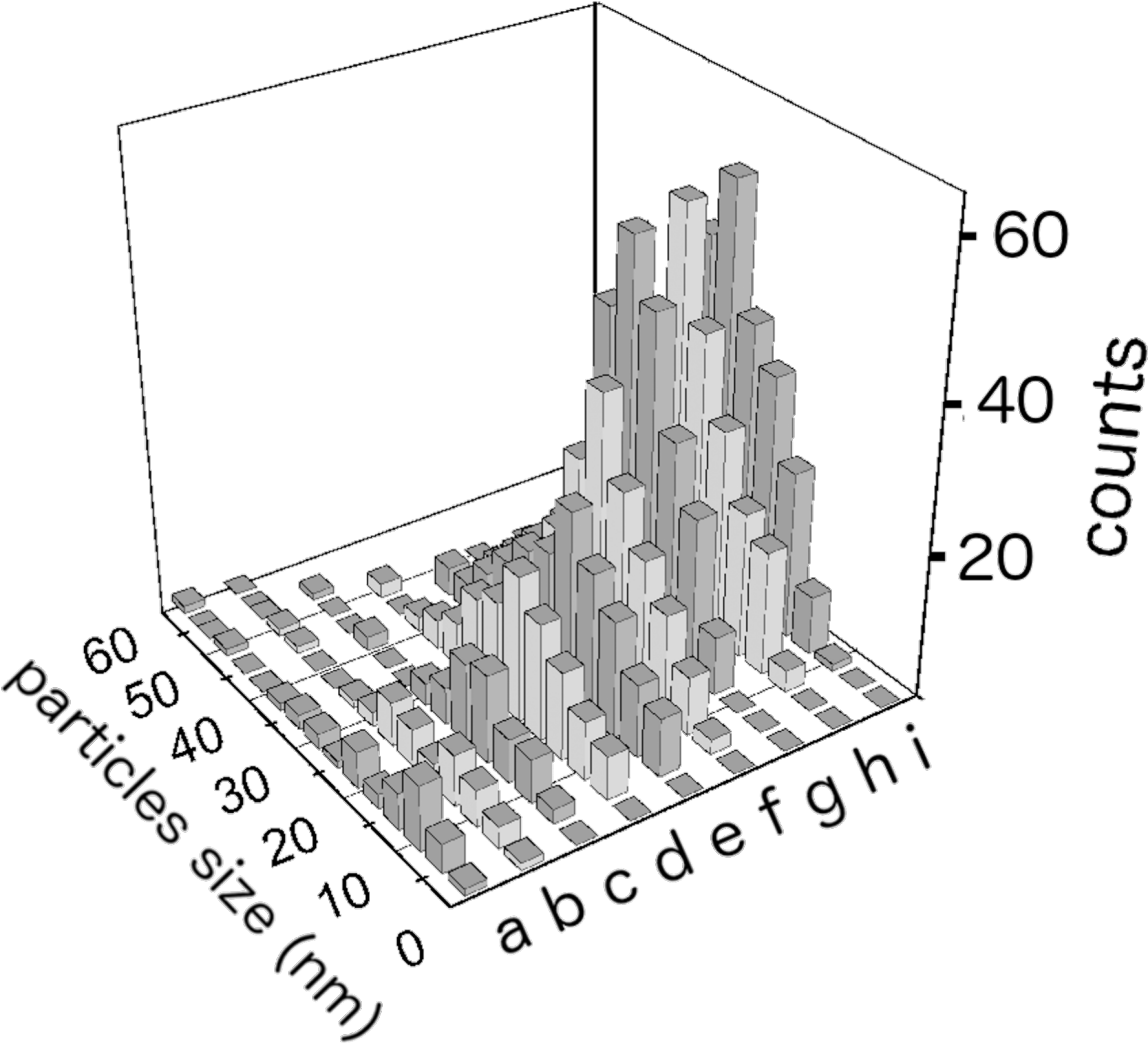
Size distribution of silver nanoparticles obtained from samples containing different NH_3_ concentrations: (a) without ammonia and (b–i) with 0.01, 0.1, 1, 5, 10, 100, 1000 and 2000 ppm ammonia.

In order to obtain statistical information and determine the nanoparticles size, TEM images, which were obtained from 50 randomly chosen areas, were processed with "Digital Micrograph" a Gatan software and the data were reported in histograms (Figure 4). In such a way, more than 200 particles were analysed for each sample and the obtained results are statistically meaningful. From the analysis of the results in Figure 4 we can see that at low ammonia concentrations (from 0 to 1 ppm) the average diameter of nanoparticles increases with concentration and its distribution evolves towards a theoretical Gaussian distribution. From 1 to 100 ppm the average size of nanoparticles does not change, but the number of nanoparticles is significantly increased. A further increase in ammonia concentration does not lead bigger numbers of nanoparticles.

Table 1 shows the mean values of the diameters <*d*> and the relative standard deviations σ, together with the coverage index defined as *R* = *S*_p_/*S*_i_, where *S*_p_ represents the fraction area covered by nanoparticles and *S*_i_ the surface of the image. The absolute value of *R* obtained from a single TEM image is meaningless. However, the average of all the images processed for each sample, and, above all, the value of *R* as a function of the ammonia concentration provide important information. In fact, the table shows that ammonia has little influence on the size of nanoparticles, but has a significant influence on their quantity.

**Table 1:** Nanoparticle mean diameter <*d*> and relative standard deviations σ together with the coverage index *R*.

NH_3_ (ppm)	<*d*> (nm)	σ (nm)	*R*

0	18	12	0.02
0.01	19	10	0.03
0.1	20	9	0.05
1	22	8	0.09
5	23	8	0.14
10	25	6	0.21
100	28	6	0.39
1000	27	6	0.38
2000	28	6	0.41

**Figure 5:**
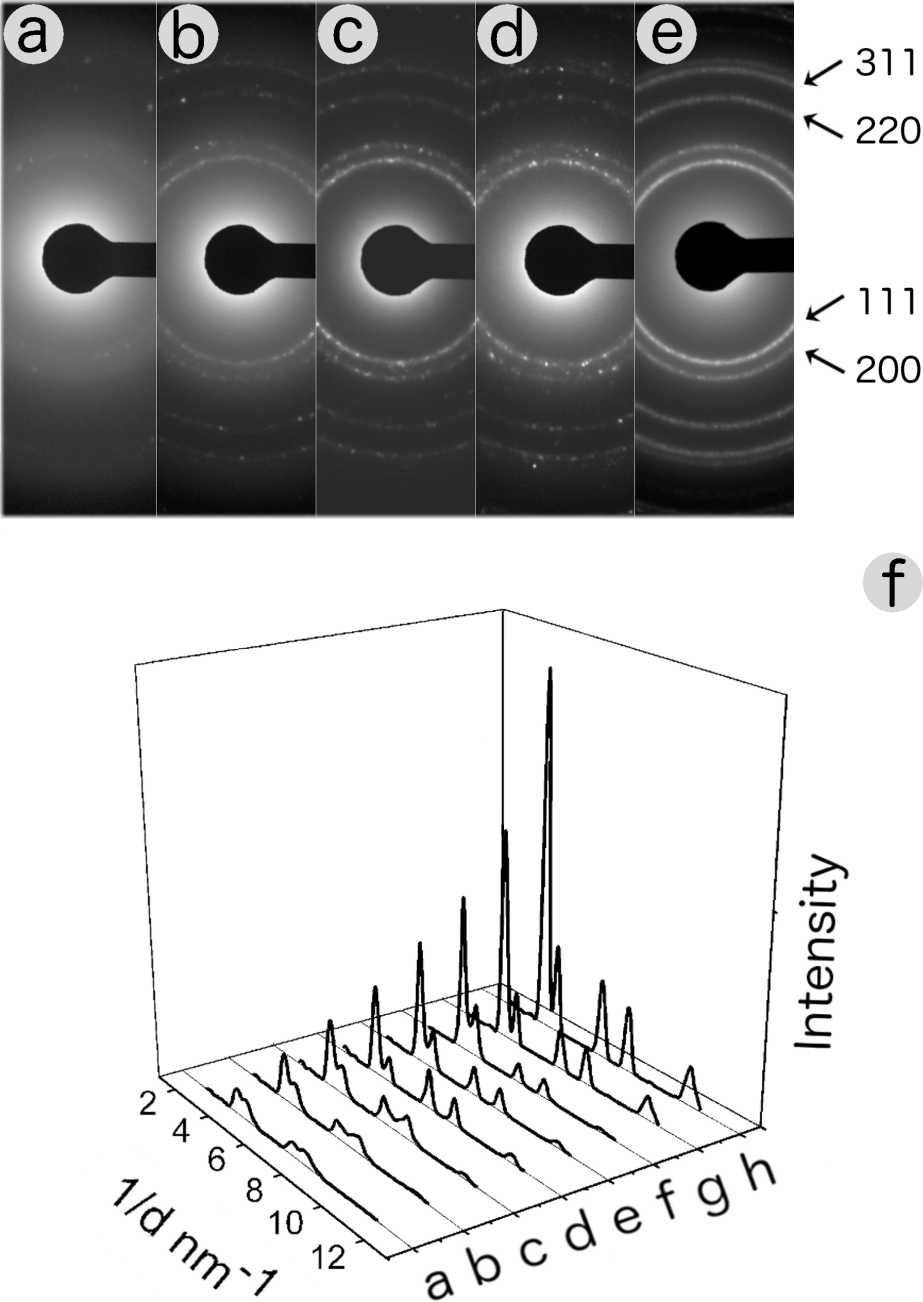
SAED patterns of the colloidal solutions obtained (a) without ammonia and (b–e) with 1, 10, 100 and 1000 ppm ammonia. Panel (f) shows the intensity of the diffraction profiles (a) without ammonia and (b–h) with 0.01, 0.1, 1, 5, 10, 100 and 1000 ppm ammonia.

Figure 5a–e shows five representative SAED patterns that summarise the evolution of typical features of silver nanoparticles coming from colloidal solutions obtained without ammonia and with 1, 10, 100 and 1000 ppm NH_3_.

The diffraction patterns provide a qualitative description of the structure of our nanoparticles: Discontinuous rings with spots and of low intensity originate from regions with a relatively small number of nanoparticles. As the nanoparticles in the selected area increase, the diffraction rings become more continuous and intense until they reach a saturation level. This information can be quantified by the PASAD-tools software: background-subtracted diffraction profiles on the entire sequence of diffraction patterns recorded from our samples are shown in Figure 5f. The profile obtained without the addition of ammonia shows very wide diffraction peaks with a very weak intensity. The addition of ammonia results in the formation of diffraction peaks that are concentration-dependent, increasingly intense and well identifiable. Until, at concentrations of around 1000 ppm, saturation is observed: The intensity of diffraction peaks remains constant.

It is possible to correlate the intensity increase of the plasmon resonance peak to the increase in the concentration of silver nanoparticles in the colloidal solutions. The increase in absorbance does not depend linearly on the concentration of NH_3_. In particular, as shown in Figure 2, at first, the absorbance increases weakly with increasing of NH_3_ concentrations up to about 0.5 ppm, then the absorbance increases more sharply as the concentration of NH_3_ goes up to about 100 ppm, then, at higher ammonia concentrations, the increase in absorbance becomes very weak. The behaviour observed in Figure 2 is determined by the morphology and number of nanoparticles. When saccharides only were used both as reducing and capping agent the obtained silver nanoparticles have lower stability and aggregation occurred. Ammonia increases the stability of AgNPs in a concentration-dependent manner.

**Figure 6:**
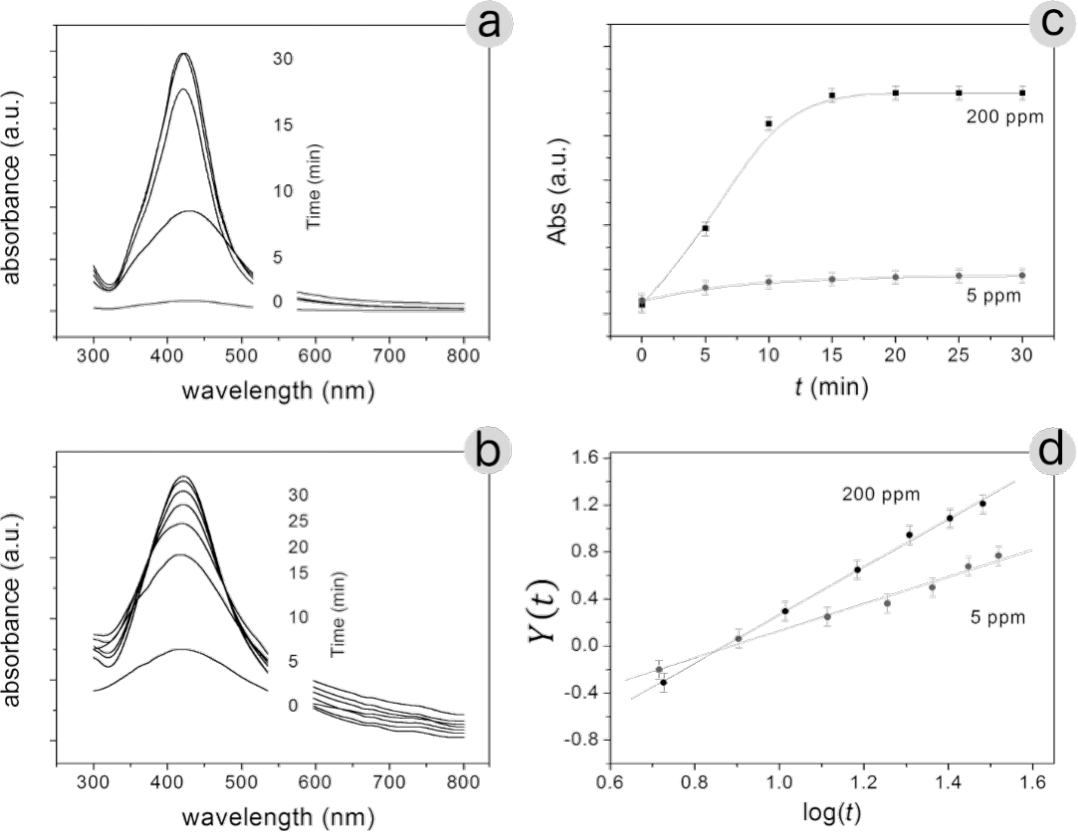
UV–vis absorption spectra recorded as a function of the reaction time with (a) 200 ppm and (b) 5 ppm ammonia; (c) SPR intensity (Abs) as a function of the reaction time; (d) experimental values of *Y*(*t*) = log{ln[abs_∞_/(abs_∞_ − abs(*t*)]} plotted and fitted according to Equation 1.

Several sets of experiments were carried out to determine the role of ammonia in the process of silver reduction. In particular, the results were interpreted by a first-order kinetic model [30–31]. As expected, the final result depends on NH_3_ concentration and on reaction time. Figure 6 shows the time evolution of UV–vis absorption spectra of two different colloidal solutions containing 200 ppm and 5 ppm ammonia (Figure 6a and Figure 6b, respectively). Figure 6c shows the SPR intensity (Abs) as a function of the reaction time for the two colloidal solutions considered.

The obtained results agree very well with the kinetic model. The absorbance (abs) of the plasmon resonance band is related to the volume fraction of the nanoparticles in the colloidal solution as a function of the reaction time (*t*):

[2]
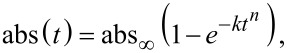


where abs_∞_ represents the absorbance saturation value, and the constants *k* and *n* are, respectively, the apparent rate constant and the Avrami exponent. Zhou et al. [32] write *n* as

[3]
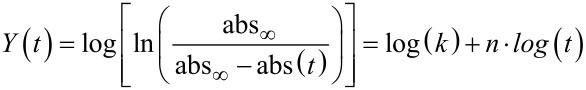


where *a* denotes the time-dependent nucleation rate (0 < *a* < 1), *b* is connected to the dimensionality of the growing particles (*b* can assume the values: 1, 2, and 3), and *c* is the growth rate in each dimension (*c* is either 0.5 or 1) [33–35].

It can be shown that

[1]
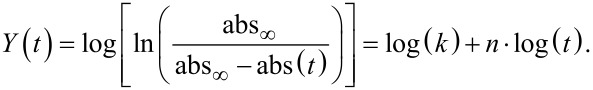


Figure 6d report the experimental values as suggest by Equation 1: *Y*(*t*) as a function of log(*t*). In this way, the constants *k* and *n* can be obtained from the line intercept with the y-axis and the slope of the straight line (Table 2).

**Table 2:** Constants *k* and *n* determined according to the proposed processing of the experimental data.

NH_3_ concentration (ppm)	*k*	*n*

5	0.125 ± 0.006	1.04 ± 0.05
200	0.0166 ± 0.008	2.05 ± 0.08

Different NH_3_ concentrations give rise to different Ag nanoparticles agglomeration and nucleation rates. In particular, the rate of nucleation of the nanoparticles depends on the concentration of ammonia, is very low in the presence of low concentrations of ammonia and increases if the concentration of ammonia increases.

**Figure 7:**
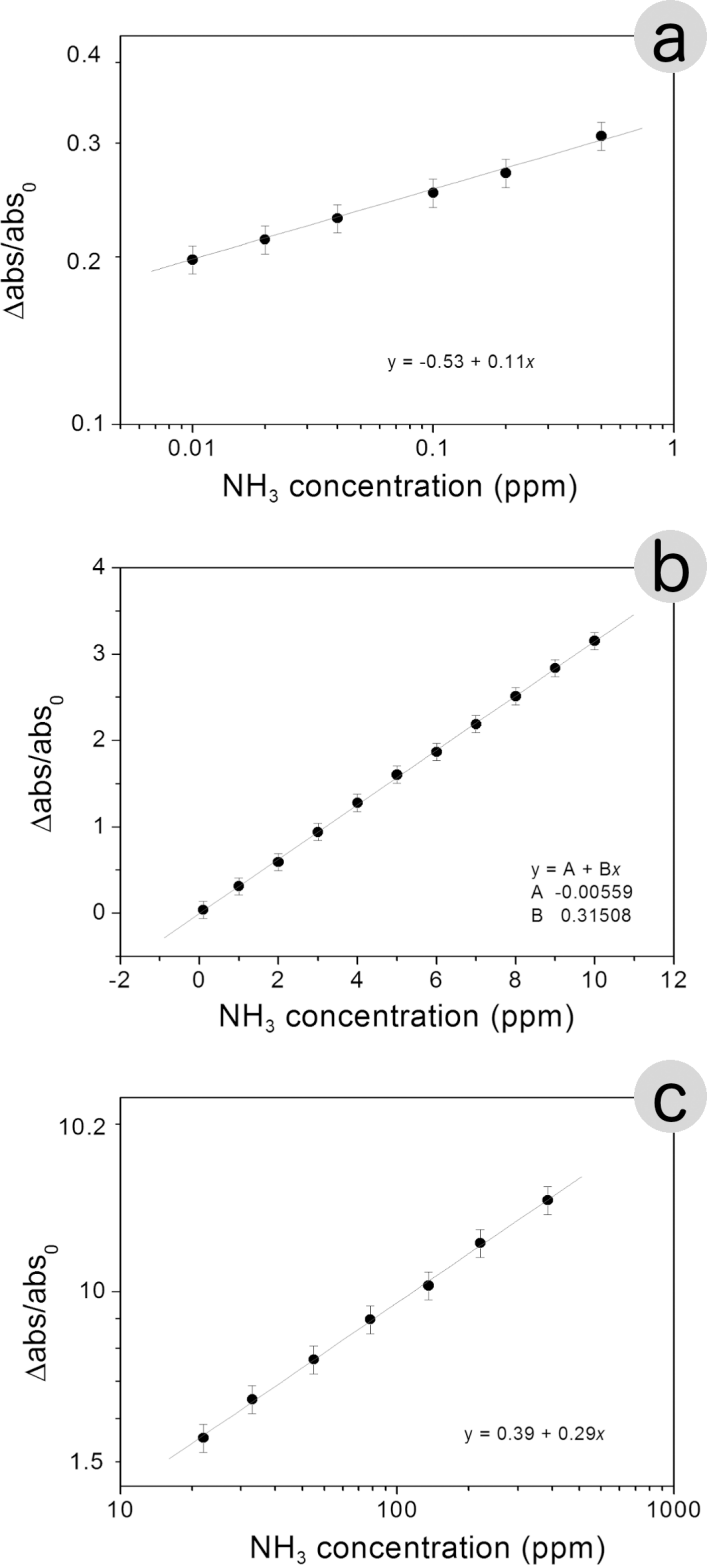
Three distinct regimes of absorbance as a function of the NH_3_ concentration: (a) very low concentrations from 0.01 to 0.2 ppm, (b) intermediate or physiological concentrations from 0.5 to 10 ppm, (c) high concentrations from 20 to 350 ppm.

The presence of ammonia affects the intensity of the plasmon resonance curve. This effect could be used to determine the presence and the concentration of ammonia in a solution. Figure 1 and Figure 2 clearly show the sensitivity of absorbance change to concentration of ammonia, over many orders of magnitude (from 10^−2^ to 10^3^ ppm). While noting that the absorbance increases as a function of the concentration of NH_3_, it is not possible to identify a unique functional dependence, valid over the whole ammonia concentration range used. It is possible to identify three distinct regimes of absorbance as a function of the NH_3_ concentration: The low ammonia concentration range from 0.01 to 0.2 ppm (Figure 7a), the intermediate or physiological range of ammonia concentrations from 0.5 to 10 ppm (Figure 7b), and the high ammonia concentration range from 20 to 350 ppm (Figure 7c). (Δ_abs_/abs_0_) = [(abs(*c*) − abs_0_)/abs_0_] represents the relative absorbance variation, where Δ_abs_ = abs(*c*) − abs_0_ is the absorbance variation, abs(*c*) is the maximum absorbance of the SPR band obtained at ammonia concentration [*c*] in the colloidal solution and abs_0_ is the maximum absorbance of SPR band without ammonia. It should be noted that the graphs of Figure 7a and Figure 7c are plotted on a logarithmic scale, while Figure 7b is a linear graph.

The maximum absorbance value versus NH_3_ concentration, can be described by the following equation in which *H* and *p* are constants:

[4]
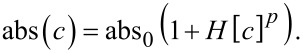


From the previous equation one can obtain:

[5]
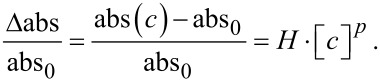


The different regimes in Figure 7 can be explained by the same function (Equation 5) with different values of *H* and *p*. According to Equation 5 the experimental points are arranged along a straight line also in the log–log plot of Figure 7a and Figure 7c. The fitting allows us to determine the values of *H* and *p* reported in Table 3.

**Table 3:** Values of *H* and *p* determined according to the proposed processing of the experimental data.

NH_3_ concentration range	*H*	*p*

0.01–0.20 ppm	0.31	1
20.0–350 ppm	2.45	0.29

The addition of small amounts of ammonia (up to 0.5 ppm) catalyse the formation of nanoparticles less rapidly and with less efficiency to what happens in the range of 20–350 ppm. Much more interesting appears to be the physiological range, where the ratio Δabs/abs_0_ is a linear function of the NH_3_ concentration (Figure 7b).

The effects of ammonia from 10^−2^ to 10^2^ ppm are measurable. Above 500 ppm, there is a saturation effect and the use of this effect to measure of ammonia in a fluid becomes unreliable. The correlation between the change in absorbance and the concentration of ammonia make the system a good candidate for the realization of ammonia sensors in the range of 0.01–350 ppm. The ammonia, added to the colloidal solutions, has the capability to control and stabilize the nanoparticles. In addition, as also observed by Nesakumar et al. the formation of the diamine silver complex increases the SPR absorbance of AgNPs in solutions containing ammonia, which increases the rate of nucleation [28]. Compared to literature results, we explore a wide range of NH_3_ concentration. See, for example, the limited explored concentration range (from 10 to 50 ppm NH_3_) in the paper of El-Sherbiny et al. [36] or until 160 ppm of Gupta and co-workers [37]. In addition, our system is able to detect low ammonia concentrations from 0.01 to 0.5 ppm.

A simple model for the interpretation of experimental results is proposed, but in many works, such as Gupta and Verma [37], only the effect of ammonia is shown. However the functional dependence of absorbance as a function of the concentration is not discussed. Others report on much higher concentrations of ammonia, such as for example the work of Detsri and Popanyasak [38], which discussed the effect of HN_3_ in a concentration varying between 10 and 800 mM, corresponding to the range from 1.8·10^2^ to 1.4·10^4^ ppm.

## Conclusion

Sucralose–glucose silver nanoparticles have been successfully synthetized in presence of ammonia in different concentrations. The optical properties of the colloidal solutions have been analysed and correlated with the morphological and structural features of nanoparticles obtained by TEM analysis. The role of ammonia in the synthesis of silver nanoparticles has been thus highlighted. The ammonia, added to the colloidal solutions, has the capability to control and stabilize the nanoparticles*.* We also observe that the kinetics of nucleation and synthesis depends on the concentration of ammonia present in the colloidal solution. The addition of ammonia changed the kinetics of the synthesis of silver nanoparticles improving oxidation of hydroxy groups in glucose, and giving rise to an accelerated reduction of silver in the fluid. The rationale of this behaviour is given by correlating the formation of nanoparticles to the content of silver ions in the solution. The concentration of silver ions is constant for all syntheses carried out in the present work. Initially, at low concentrations of ammonia, the oxidation of hydroxy groups facilitates the rate of nucleation of silver nanoparticles. Then a high rate of nucleation of silver nanoparticles was observed in the NH_3_ concentration range of 0.5–200 ppm. Finally, the silver ions run out and the rate of nucleation goes into saturation.

## Experimental

### Materials

Silver nitrate (AgNO_3_, 99%), α-D-glucose (C_6_H_12_C_6_, 99.99%), sucralose (C_12_H_19_Cl_3_O_8_, 98%) and ammonia (30% solution) were purchased from Sigma-Aldrich and used without further purification. During the experiments, deionized ultrafiltered Milli-Q grade water was used. Several ammonia concentrations were prepared by suitable dilutions in ultrapure water.

### Protocol for ammonia sensing

The synthesis of the colloidal AgNP seed solution represents the first step in the detection of ammonia. The seeds of AgNPs have been prepared using an environmentally friendly hydrothermal method that uses sucralose as a stabilizer and glucose as a reducing agent. Briefly, 2 g of α-D-glucose and 2 g of sucralose were dissolved in 100 mL of ultrapure water. The solution is heated to 90 °C, then 2.5 mL of an aqueous AgNO_3_ solution (*c* = 10^−2^ M) is added and the solution is maintained at this temperature for 5 min until it becomes pale yellow, which is indicative for the formation of AgNPs seeds. The colloidal solution with newly formed seeds is not stable. It is necessary to reduce the temperature to 50 °C abruptly to block the formation of silver nanoparticles. The obtained colloidal solution was given into 25 mL vials. Finally, ammonia was added to each vial in known concentrations ranging from 0 to 2000 ppm. The vials containing ammonia were heated and maintained at 90 °C for 35 min. In all experiments, ammonia was added to colloidal solutions within 60 min after seed formation. The final colloidal solution is very stable:The optical absorption curves can be reproduced without any significant differences for at least five days.

### Spectroscopic and morphologic characterisations

Colloidal silver nanoparticles were monitored at different reaction times (from 0 until saturation) and at different concentrations of ammonia. Aqueous solutions containing AgNO_3_ and increasing ammonia concentrations were used as references. A Varian Cary 5 spectrophotometer with a quartz cuvette of 10 mm path length was used to acquire absorption spectra of all colloidal silver nanoparticle solutions in the range of 300–800 nm spectral range. All measurements were performed at room temperature. All obtained silver nanoparticle solutions were prepared for TEM observations by casting single drops onto standard carbon supported 600-mesh copper grids and drying slowly in air. A transmission electron microscope Hitachi 7700, operating at 100 kV, was used to record TEM images. SAED figures have all been obtained by selecting areas with 5 μm diameter on the sample. The acquisition time was suitable to avoid saturation of the more intense reflections. Statistical information on TEM images was obtained by processing the images using "Digital Micrograph", a Gatan software. The diffraction patterns were processed by PASAD-tools [39] from Digital Micrograph.
